# Feasibility of using KardiaMobile-L6 for QT interval monitoring during the early phase of the COVID-19 pandemic in critical care patients

**DOI:** 10.1038/s41598-023-37688-8

**Published:** 2023-07-06

**Authors:** Matilde Zaballos, Ignacio Fernández, Lucia Rodríguez, Silvia Orozco, Amparo García, Mónica Juncos, Sara Alvarez-Zaballos, Patricia Piñeiro, Javier Hortal

**Affiliations:** 1 Department of Forensic Medicine, Psychiatry and Pathology, Complutense University, C/ Dr Esquerdo nº46, 28007 Madrid, Spain; 2 Department of Anaesthesiology, Hospital General Universitario Gregorio Marañón, Madrid, Spain; 3 Department of Cardiology, Hospital General Universitario Gregorio Marañón, Madrid, Spain; 4 Department of Pharmacology, Complutense University, Madrid, Spain

**Keywords:** Cardiovascular diseases, Infectious diseases

## Abstract

The electrocardiogram (ECG) represents an essential tool to determine cardiac electrical abnormalities in COVID-19 patients, the effects of anti-SARS-CoV-2 drugs, and potential drug interactions. Smartphone-based heart monitors have increased the spectrum of ECG monitoring however, we are not aware of its reliability in critically ill COVID-19 patients. We aim to evaluate the feasibility and reliability of nurse-performed smartphone electrocardiography for QT interval monitoring in critically ill COVID-19 patients using KardiaMobile-6L compared with the standard 12-lead ECG. An observational comparative study was conducted comparing consecutive KardiaMobile-6L and 12-lead ECG recordings obtained from 20 patients admitted to the intensive care unit with SARS-CoV-2 infection and on invasive mechanical ventilation. The heart rate-corrected QT (QTc) intervals measured by KardiaMobile-6L and 12-lead ECG were compared. In 60 percent of the recordings, QTc intervals measured by KardiaMobile-6L matched those by 12-lead ECG. The QTc intervals measured by KardiaMobile-6 and 12-lead ECG were 428 ± 45 ms and 425 ± 35 ms (*p* = 0.82), respectively. The former demonstrated good agreement (bias = 2.9 ms; standard deviation of bias = 29.6 ms) with the latter, using the Bland–Altman method of measurement agreement. In all but one recording, KardiaMobile-6L demonstrated QTc prolongation. QTc interval monitoring with KardiaMobile-6L in critically ill COVID-19 patients was feasible and demonstrated reliability comparable to the standard 12-lead ECG.

## Introduction

The disease caused by the novel severe acute respiratory syndrome coronavirus 2 (SARS-CoV-2), named coronavirus disease 2019 (COVID-19), can generate mild, moderate, severe, and critical (i.e., requiring intensive care unit [ICU] admission) disease^1–3^.

During the first wave of the COVID-19 pandemic, due to the urgency for clinical management of infected patients in the ICU, patients received anti-SARS-CoV-2 drugs with potentially adverse cardiovascular effects, such as QT prolongation^4^. The drugs hydroxychloroquine, azithromycin, and lopinavir/ritonavir were frequently prescribed; these drugs are known to prolong the QT interval and promote ventricular arrhythmias. In addition, pre-existing cardiovascular disease, inherited arrhythmic syndrome (Brugada syndrome, long QT syndrome, and hypertrophic cardiomyopathy), and systemic inflammation associated with COVID-19 can directly affect the cardiovascular system and increase the risk of arrythmias^5–7^.

In this context, the electrocardiogram (ECG) represents an essential tool to determine cardiac electric conduction abnormalities in COVID-19 patients, the effects of anti-SARS-CoV-2 drugs, and potential drug interactions^8^. In routine practice, patients admitted to the ICU or coronary units may require ECG monitoring including QT interval evaluation. This is particularly relevant for COVID-19 patients due to the therapeutic options available at the time and the additive QT prolonging effects of treatments administered during their ICU stay^9,10^.

Traditionally, the QTc interval is calculated using specific leads of a 12-lead ECG^11^. The acquisition of a patient’s QTc interval in this way requires additional nurse personnel to be exposed to equipment provided with multiple ECG wires, increasing the potential transmission of COVID-19 and other contagious diseases^12,13^. While some bedside monitors can use a 5-lead system to measure the QTc interval continuously, in many resource-limited intensive care units this is not possible. In addition, the tracing is typically not recorded and cannot be printed, limiting its usefulness in certain contexts where one wants to review the temporal evolution of the QTc interval with the treatments received by the patients. Moreover, although the QT interval can be measured automatically, the experts advocate manual assessments.

In response, several smartphone-based heart monitors and their corresponding mobile applications have recently been developed, which has increased the spectrum of ECG monitoring to include accurate QT interval measurements^14^.

During the COVID-19 pandemic, the United States Food and Drug Administration (FDA) approved KardiaMobile 6L (AliveCor Inc., United States), a device for QTc monitoring in COVID-19 patients^15^. This development has enabled the assessment and monitoring of COVID-19 patients’ QT intervals at home in addition to the hospital^16,17^. However, to date, there is no study comparing the accuracy of QT interval measurements in critically ill patients using the standard 12-lead ECG and using a smartphone device.

The primary aim of the present study is to evaluate the feasibility and reliability of nurse-performed smartphone electrocardiography for QT interval monitoring in critically ill COVID-19 patients using KardiaMobile 6L compared with the standard 12-lead ECG.

## Methods

### Study design

The study was conducted using an observational comparative design. Ethical approval (13/2020) was obtained from the Human Ethics Committee of the Hospital Universitario Gregorio Marañón, Madrid, Spain (Chair F. Atienza-Fernández) on 7 May 2020, which waived patient consent for this evaluation because of the observational study design. The methods were carried out in accordance with the relevant guidelines and regulations.

### Participants

Patients included in this study were admitted to the ICU with confirmed SARS-CoV-2 infection and one of the following characteristics: respiratory failure requiring mechanical ventilation, shock, and organ failure requiring intensive care therapy. The sample only includes a subgroup of patients who were admitted between March 2020 and May 2020.

### Measurements and data collection

In accordance with hospital protocol, all patients admitted to the ICU undergo an ECG. To evaluate how KardiaMobile 6L can enable QT interval monitoring in critically ill patients, 20 patients underwent consecutive recordings, first with the KardiaMobile device, then with the standard 12-lead ECG.

**Figure1:**
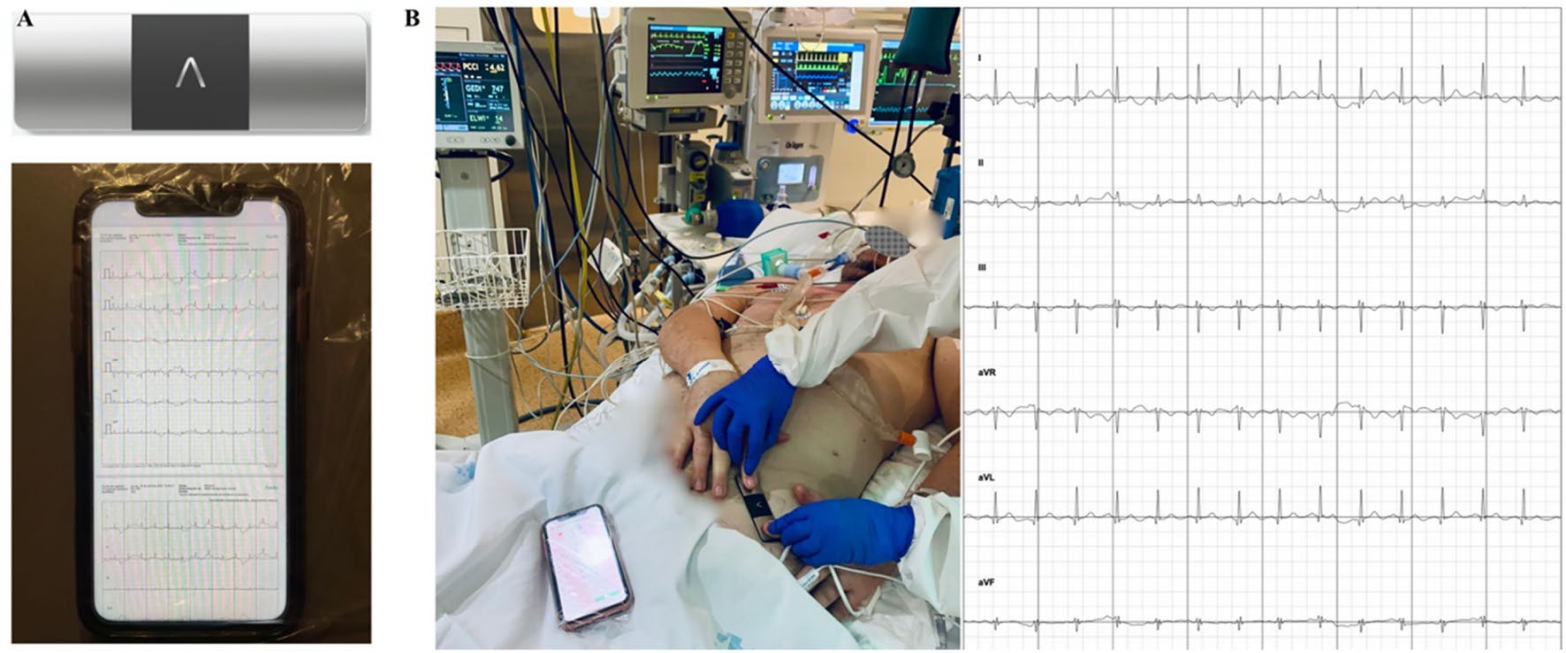
(**A**) The KardiaMobile and the smartphone placed in a biohazard bag. (**B**) ECG recording with KardiaMobile in a COVID patient on mechanical ventilation in the ICU.

KardiaMobile 6L records six of the 12 leads of a standard ECG, which allows the device to be used for QT/QTc interval monitoring. The device has several theoretical advantages over the standard 12-lead ECG: simplicity of use, small size, remote data transmission (i.e., minimized risk of contamination), and easy disinfection. Using the device, short ECG recordings can be obtained; by reducing monitoring time, the other needs of the patient can be attended to. The ECG recording is transmitted to a receiving system (smartphone) connected via Bluetooth to the KardiaMobile device (Fig. 1).

To register a 6-lead ECG with KardiaMobile, the ECG is placed on the patient’s bare skin at the root of their left thigh; the healthcare provider then holds the patient's fingers of both hands together on top of the device’s electrodes for 30 s (Fig. 1). The device is cleaned by spraying it with an alcohol-based disinfectant and wiping it with a soft cloth, which must be done before it can be used on another patient.

In the present study, an ECG from a 12-lead ECG was obtained immediately after one from KardiaMobile L6, reflecting common clinical practice.

### ECG analysis

**Figure 2:**
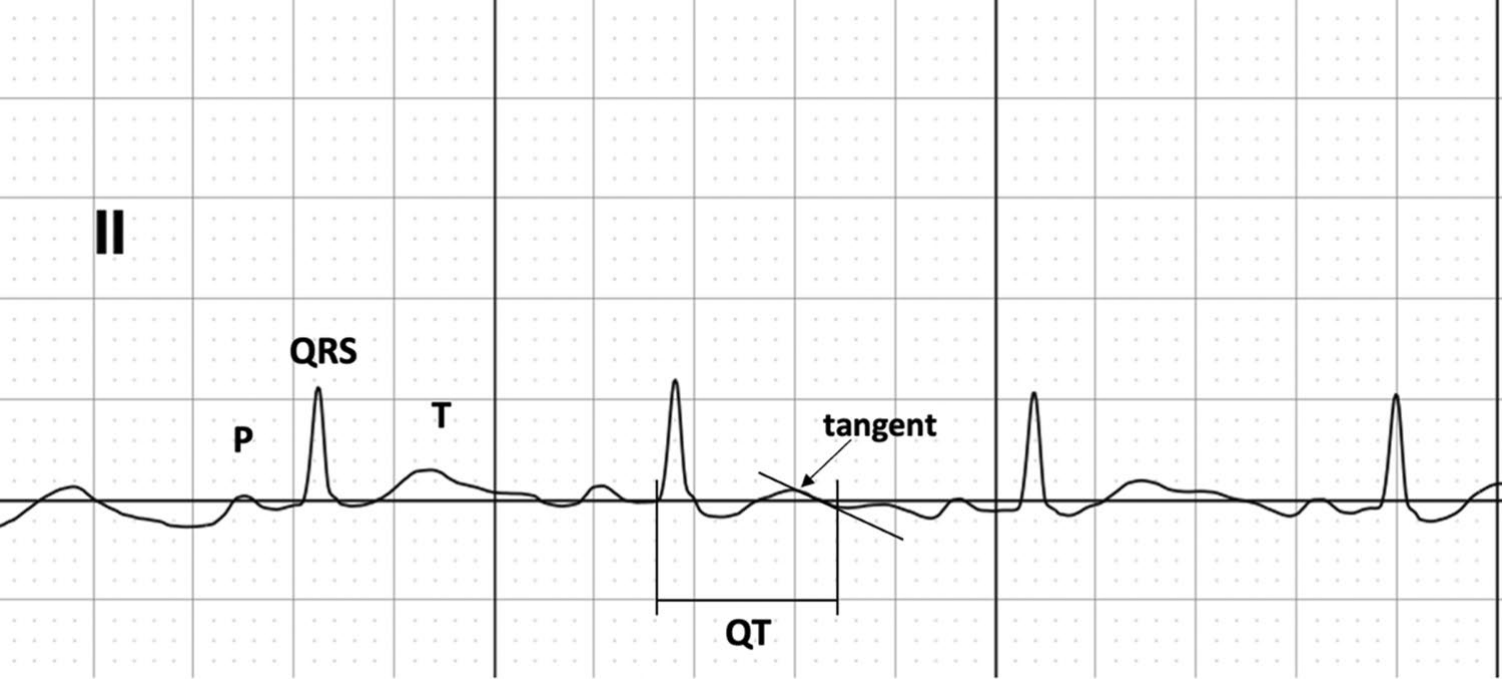
Illustration of QT interval measurement.

All acquisitions were performed and supervised by study investigators. QT and QTc intervals were calculated based on recommendations in the literature^11,18^. The Bazett method was used to correct the QT interval for heart rate (QTc = QT/RR^1/2^). In brief, when the T wave morphology was normal, the T wave offset was at when the descending limb returned to the T–P baseline (Fig. 2). To ensure data integrity, the KardiaMobile recordings were compared to the 12-lead ECG recordings by two independent physician reviewers.

### Statistical analysis

The analysis was conducted using IBM SPSS Statistics on a Mac (IBM Corp., Version 27.0. Armonk, NY). The results are presented as medians and interquartile ranges (IQR) for non-normally distributed data, using the Shapiro–Wilk test, and as means ± standard deviation for normally distributed variables. Categorical variables were described as percentages. Either a Student’s t-test or Mann–Whitney *U* test was used for testing differences between continuous variables for each device, while differences between categorical variables were tested with the Fisher test.

A comparison of QT interval measurements between 12-lead ECG and KardiaMobile was performed using the Bland–Altman method of measurement agreement. In addition, Pearson correlations were calculated for QTc interval measurements between 12-lead ECG and KardiaMobile. Furthermore, to report the correlation between the two measurements of QT intervals, we used the intraclass correlation coefficient (ICC) and corresponding 95% confidence interval (CI). A *p* value below 0.05 was regarded as statistically significant.

### Simple size

There are no studies in intensive care patients in which the KardiaMobile has been used to measure the QT interval. A previous study showed that KardiaMobile detected the QT effects in 20 patients undergoing treatment with drugs known to prolong the QT interval^14^.

## Results

The clinical characteristics of the patients are summarized in Table 1.

**Table 1:** Clinical Characteristics of COVID patients.

Number of patients	20
Age (years)	63 ± 13
Male (%)	80
Female (%)	20
Invasive mechanical ventilation (%)	100
Hypertension (%)	65
Diabetes (%)	25
Obesity (%)	55
Cardiovascular disease (%)	30

Data are showed as means ± standard deviation and percentage.

At the time of ECG determination, all the patients were on invasive mechanical ventilation, and the predominant pathologies were hypertension and obesity. Three and two recordings were repeated with KardiaMobile and 12-lead ECG, respectively, due to the presence of artefacts.

The QTc intervals were 428 ± 45 ms and 425 ± 35 ms (*p* = 0.82) measured by 12-lead ECG and KardiaMobile, respectively. Table 2 shows the ECG parameters that were analyzed. Forty percent of the patients had a QTc interval above the limit (470 ms in men and 480 ms in women), while two patients had an interval above 500 ms.

**Table 2:** Results from the ECG data.

	12-Lead ECG	KardiaMobile	*P* value
Heart rate	86 (75–100)	91 (79–100)	0.51
PR interval (msec)	140 (120–160)	150 (120–160)	0.91
QRS duration (msec)	80 (60–80)	70 (60–80)	0.77
QTc (msec)	428 ± 45	425 ± 35	0.82

Data are showed as means ± standard deviation for normally distributed variables and as medians (IQR) for non-normally distributed data using the Shapiro–Wilk test.

Thirty-five percent of the patients were treated with at least two drugs with QT prolonging effects; in this subgroup, patients’ QTc intervals were not longer than those of patients who were not treated with QT-prolonging drugs: 429 ± 38 ms versus 426 ± 40 ms (*p* = 0.87). In all but one recording, KardiaMobile detected the presence of a QTc interval when greater than 450 ms. None of the patients evaluated had arrhythmias during their ICU stay.

**Figure 3:**
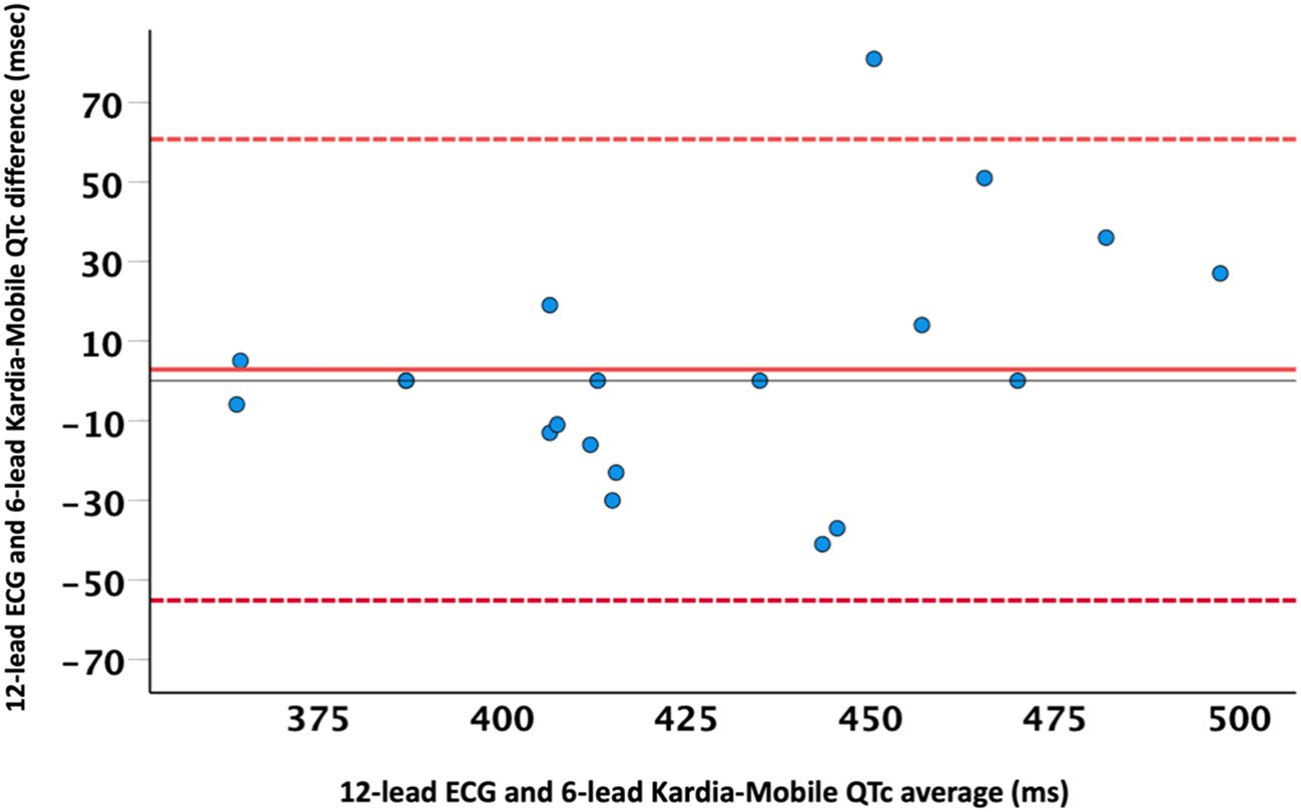
Comparison of QTc intervals between KardiaMobile and standard 12-lead ECG measurements using the Bland–Altman method for analysis of measurement agreement. Solid and dotted lines represent the difference and 95% confidence interval of the difference in QTc interval between the 2 methods, respectively.

QTc intervals measured by KardiaMobile demonstrated good agreement (bias = 2.9 ms; standard deviation of bias = 29.6 ms) with 12-lead ECG, using the Bland–Altman method of measurement agreement. The correlation coefficient of QTc intervals between the two devices was 0.75 (*p* = 0.001). The ICC between the two measurements of QTc intervals was 0.84 (95% CI [0.6–0.93]; *p* < 0.001) Fig. 3.

In total, 60 percent of the recordings showed a difference of less than 20 ms between the two devices.

## Discussion

The main findings of the present study are (1) KardiaMobile L6 has shown feasibility and reliability in measuring the QT interval in critically ill COVID-19 patients compared to the standard 12-lead ECG and (2) the device was capable of detecting QTc prolongation in the same. These two findings suggest that KardiaMobile may be a suitable device for QTc interval monitoring and to detect electrical cardiac abnormality in critically ill COVID-19 patients.

Several existing reports suggest that SARS-CoV-2 infection can result in cardiovascular complications and exacerbate pre-existing cardiovascular disease^5–7^. Similarly, many drugs employed for treating COVID-19 may prolong the QT interval, which can lead to the development of polymorphic ventricular tachycardia (VT) in the form of torsade des pointes (TdP)^4,5^. Therefore, the ECG represents an essential tool for surveillance and early detection of cardiac electrical abnormalities in order to alert clinicians to the presence of patients at risk.

Nurses in the intensive care unit faced a considerable higher risk of biological exposure during the recent coronavirus disease pandemic^19^. As a result, numerous initiatives were developed to maintain the quality and safety of care for patients while reducing the risk for the professionals caring for them. An important advantage of KardiaMobile L6 over the standard 12-lead ECG is that patients can obtain an ECG themselves; provided that patients are capable of cooperating and training requires only 1–2 min^20,21^. While such training is not practical in a critical care setting, where an ECG is performed by a healthcare professional, the simplicity of KardiaMobile compared to 12-lead ECG saves time and reduces the risk of transmission for nurses in the ICU. In addition, 12-lead ECG equipment must be thoroughly cleaned and disinfected before and after each use, which requires more time and attention than the disinfection of the KardiaMobile device. This consideration could apply in any pandemic requiring respiratory isolation and in overcrowded hospitals with limited availability of healthcare professionals and protective equipment.

In previous studies evaluating the usefulness of KardiaMobile for QT interval monitoring in COVID-19 patients, patients admitted to the ICU were excluded^21–23^.

González et al., recorded the QT interval in 81 hospitalized patients with confirmed SARS-CoV-2 infection who were being treated with drugs with a known risk of QT prolongation^21^. The authors validated their 12-lead ECG and KardiaMobile recordings in a control group. In their study, KardiaMobile was found to be effective in detecting QT prolongation, which allowed for treatment adjustments in the affected patients. While they used the same KardiaMobile device as the present study, they simplified the recording to a single lead by placing the device on the anterior aspect of the left thorax, which is below the nipple in men and under the breast in women, close to the apex of the heart. In this position, performing an ECG is easier for nurses and does not require the patient’s cooperation. We instead opted for a 6-lead recording, despite these benefits, because it is considered more appropriate for studying the QT interval; single-lead measurements provide only a fraction of the information available from multi-lead analysis^11,13^. In this way, we aimed to improve upon González et al.’s study by obtaining better information on critically ill patients with more severe SARS-CoV-2 infection^21^.

In a similar study, Minguito-Carazo et al. recorded the QT interval using KardiaMobile L6 in hospitalized patients who performed the ECG themselves^22^. The authors evaluated the correlation between the 12-lead ECG and KardiaMobile recordings in a subgroup of healthy patients. The intraclass correlation showed a moderate reliability of 0.56; notably, this is lower than the 0.87 shown in the present study. The correlation observed in this study may be better because the ECG recordings in our patients were performed by nurses or physicians, as opposed to the patients themselves, which would foreseeably affect the quality of the recording. The authors also reported that it took significantly longer to record a 12-lead ECG compared to an ECG from KardiaMobile in COVID-19 patients: 519.0 ± 94.1 versus 107.1 s. In the present study, we did not measure recording time with either device, although the results are expected to be similar to those reported in Minguito-Carazo et al.’s study^22^.

In our sample, three of the KardiaMobile recordings had minor artifacts, which made it difficult to read the QT interval, while two of the 12-lead ECG recordings were profoundly affected by artifacts. We reason that, among other factors, the patient’s specific conditions in the ICU, including significant electrical noise, mechanical ventilation, and spontaneous movements of the patient, caused these artifacts.

## Limitations

Although the QT interval can be measured automatically with some bedside monitors; however, the experts advocate manual assessments because most automated systems do not routinely display the superimposed tracings, or the points used to derive the QT interval. The present study included a limited number of patients. The small sample size may not be significant, however, as we observed good agreement with and made similar findings to other studies on hospitalized patients with SARS-CoV-2 infection. We did not measure recording time for performing an ECG with either device; notably, recording time might have demonstrated an additional advantage of KardiaMobile L6 in an intensive care setting.

## Conclusions

QT interval monitoring with KardiaMobile 6L in critically ill COVID-19 patients was feasible and demonstrated reliability comparable with the standard 12-lead ECG. The device offers potentially reduced risk of transmission for staff in the ICU and potential time savings, both of which are important advantages for healthcare professionals in the current context.

## Data Availability

The data that support the findings of this study are available from the corresponding author upon reasonable request.
